# An Account of the Wounded on Board His Majesty's Ship Queen Charlotte, in the Battle of Algiers

**Published:** 1819-04-01

**Authors:** A. Dewar

**Affiliations:** Physician of the Fleet, then Surgeon of the Queen Charlotte


### Medico-Chirurgical Journal;

OR,

### Quarterly Register

OF

### MEDICAL AND SURGICAL SCIENCE.

No. 4, New Series.] APRIL 1, 1819. [Vol. I.

### FIRST DEPARTMENT.

### PRACTICAL ESSAYS AND COMMUNICATIONS,
*Original or Translated.*

### I.

### *An Account of the Wounded on Board His Majesty's Ship Queen Charlotte, in the Battle of Algiers.*

By Dr. A. Dewar, Physician of the Fleet, then Surgeon of the Queen Charlotte.

——— Factorum est copia Nobis,
Res gestæ, Regumque, Ducumque, et tristia Bella.

IN the expedition lately sent against the Algerines, I was surgeon of His Majesty's ship Queen Charlotte, bearing the flag of the Commander in Chief. The number of wounded on board that ship, in the memorable and sanguinary battle fought before Algiers, was great, and many cases of much interest occurred.—I have thought a general account of the nature and treatment of some of the more important wounds which I then treated, might not be unworthy the attention of the surgical reader; and that the facts detailed might not be destitute of interest to the profession at large, nor incapable of useful application in private practice.

The number wounded on board the flag ship amounted to 131. Of these, several were, of course, only slight; but a considerable number of them were severe and important. The success which attended the treatment of these cases was unusually great. All the wounded were kept on board until our arrival in England, about six weeks after the action, and one only died during that time; and from a cause which could not have been foreseen, and at a time when every thing promised a more favourable termination than could have been expected, from the nature of the injury, and the complication of disease under which this patient laboured, independent of the wounds. *See Case No.* 1.

The great source of danger in the first stage of wounds, inflicted on such occasions, on men generally in the prime of life, and full vigour of health, almost entirely arises from the high degree of inflammatory action which must be expected to take place, unless sufficiently active measures are employed to restrain it. To this cause, I think, may often be attributed the hæmorrhages and sloughing after capital operations; and perhaps the tetanic, and other spasmodic affections, which often follow wounds. The primary treatment of the wounded, on this occasion, was wholly directed to counteract this tendency, and the successful issue of the cases may, in some degree at least, be attributed to the active measures employed.

Some of the cases in the following pages will show to what extent bleeding may be carried in wounds of the great cavities, with safety; and what success may be expected from this treatment, energetically pursued, in cases even of very unpromising aspect. I need scarcely mention, that every other part of the antiphlogistic plan was, at the same time, rigidly enforced.

Having premised these observations, I shall now proceed to the detail of some of the cases, and shall afterwards add a few remarks, arising from the amputations which were performed.

Case 1. Aug. 28th. Lieutenant J. F. I. ætatis 24. Some hours after the action had commenced, he received a wound on the left side of the face from a splinter. The cheek was divided to the extent of about an inch and a half. This wound being dressed, he immediately returned to his quarters. A short time afterwards, he was brought down wounded a second time, and very severely. He had been struck on the left arm and side by a large splinter of a bar of iron, which carried away a portion of the pectoral muscle; the soft parts of the side, as low as the eighth or ninth rib, and as far back as the costa of the scapula. The axillary plexus of vessels and nerves was torn asunder; the os humeri was shattered into numerous portions, immediately below its neck; and the soft parts, on the fore part of the shoulder, and the posterior fold of the arm-pit, were entirely destroyed. The shattered arm was attached by a narrow slip of integuments on the back part of the arm. A small triangular portion of the back part of the deltoid muscle remained uninjured. The divided axillary artery projected beyond the surface of the wound, and poured out a slender stream of blood: this was instantly secured. The loss of blood, immediately on the receipt of the wound, was very considerable; the pulse was extremely small and rapid, and the countenance of a ghastly paleness. Some wine was administered. His mind, however, appeared unshaken; and, sensible of his dangerous situation, he expressed a conviction that nothing could be done to save him, and said, he only wished to speak with me before he died. On encouraging him with a hope that he might be saved, he immediately consented to the removal of the shattered limb. The portion of the deltoid muscle was raised from the head of the bone. Two small vessels were divided, which were immediately secured; the capsular ligament was then opened, and the head of the bone removed by cutting through the joint downwards. The soft parts in the axilla were divided about an inch above the torn extremities of the axillary plexus. A gush of blood immediately followed the division of the axillary artery; as the pressure applied above the clavicle to restrain the flow of blood had little effect, partly in consequence of the clavicle having been formerly fractured, immediately over the course of the artery, and irregularly united, it was immediately laid hold of, separated from the surrounding nerves, and secured with a ligature. Numerous splinters of bone, and ragged portions of muscle, were now removed from the surface of the wound. The small portion of deltoid left being sufficient to cover the glenoid cavity, it was brought over, and two ligatures were passed, to give support to this part. A wound of frightful extent remained uncovered. Adhesive straps were applied, to give support to the parts, and a large anodyne was administered. He passed the night quietly; considerable oozing of blood from the wounded surface. Complains of stiffness of the parts; pulse 126, soft and tremulous. Has taken a little wine and sago occasionally. Rep. haust anod. Appl. aq. frigid. parti vulneratæ.

29th. Slept well, and says he feels better this morning; pulse 124, very soft; no stool since the injury. Injic. enema simplex statim. Cont. haust. anod. h. s. et applicet. aqua frigida.

30th. Three copious stools from the injection; removed the outer bandage this morning; slept well during the night, and is cheerful. Wound tolerably easy; pulse 124, but fuller; skin warm; tongue white; thirst; generally lies as if asleep, unless when spoken to. Aq. supertart. potass. pro potu.

31st. Removed the dressings this morning. The flap has a healthy appearance externally. The exposed parts are covered with sloughs. The sutures were removed, as they appeared to have little effect. Ate some chicken broth yesterday, and occasionally a little sago. Pulse about 120; skin natural; tongue white; thirst. Bibat. potum supertart. potass. ad libit. Rep. alia.

September 1st. Passed a good night; two stools from an injection last night. Removed some sloughs from the wound this morning. The axillary artery is seen pulsating strongly in the upper part of the wound. Purulent discharge from the under part of the wound. Pulse 120, soft; skin cool; has taken chicken and mutton broth frequently yesterday and to-day. Is cheerful, and says he feels comfortable and easy. Dressed the parts with unguentum resinæ; and over all, adhesive straps as before. Cont. potus et rep. haust. anod. vespere.

2d. Passed another quiet night. Discharge of pus from under the flap. Sloughs continue to cover the surface of the wound. The pulsation of the axillary artery strong. Has been teased to-day with frequent cough, and copious expectoration. Pulse 116, and soft; skin warm; takes chicken broth frequently, and occasionally sago or tapioca; had three stools after an injection. Rep. omnia. Sorbeat mistur. mucilag. pauxill. subinde.

3d. Sloughs separating, and healthy granulations appearing on many parts of the wound. Slept well; pulse 104, soft; little cough during the night; tongue clean and moist. Rep. omnia.

4th. Was more restless yesterday afternoon, with increased heat of skin. He was frequently sponged with cold water and vinegar. Towards evening he became quiet, and passed a good night. Pulse this morning 104; sloughs separating; one ligature came away to-day. Has taken soup frequently, and eats it with a relish. Complains of slight sore throat. Fricentur fauces externæ liniment. ammoniat. et tegantur panno laneo. Cont. omnia.

5th. Passed a good night; pulse this morning 96; copious good discharge from the wound, and a great part of the sloughs are thrown off. Pulsation of the artery less distinct; takes liquid food frequently. Cont. omnia.

6th. Frequent singultus during the night; the sloughs are entirely thrown off, except from a small spot on the fore part of the acromion. Discharge good, and in moderate quantity. Pulsation of the axillary artery less distinct; the ligature remains firmly attached; had an exacerbation of fever again yesterday afternoon, which subsided about six o'clock.

7th. Frequent cough, which distresses him considerably; copious expectoration; wound healthy; ligature firmly attached; pulsation less distinct; pulse 96; skin moderately warm; one stool this morning; appetite tolerable.

℞. Mucilagin. gummi acaciæ ℥viij; tinct. op. ʒj; Sacchari ℥ß. M. Capt. semiunciam subinde tussi urgente. Rept. alia.

8th. Last night passed about four ounces of purulent-looking matter, mixed with blood by stool. Has felt no pain in any part of the abdomen; cough continues troublesome; expectoration free; pulse last night 108; this morning 104. The wound is healthy in appearance. No stool since the purulent matter passed last night.

℞. Pulv. rhei gr. x; carbonat. magnes. ʒß; aq. menth. pip. ℥j ft. haust. statim sumendus.

*Vespere.* Si opus sit, injiciatur enema. Rep. alia.

9th. Had one stool yesterday, with a considerable quantity of purulent matter; and one this morning, with a smaller quantity. The wound is healthy, and contracting at the upper part. Ligature remains attached. Pulse 92; cough troublesome. Cont. omnia. Habeat vin. rubr. ℥iv in die.

10th. A copious stool this morning, without purulent matter; cough frequent; the ligature came away this morning; perspired frequently from the upper part of the body during the night; pulse 96. Cont.

19th. The appearance of the wound has continued healthy, and its surface is considerably diminished by the action of the adhesive straps. He has continued to pass purulent matter by stool occasionally, in greater or less quantity. It appears he has now and again passed purulent matter by stool for two years past, after an attack of dysentery, which he experienced at Java. His appetite is tolerable, his appearance cheerful, and he gains strength. Pulse, towards evening, about 100; in the morning 84. Cont. med.

24th. Has been restless since midnight, although he makes no complaint of pain. Appearance of the wound continues healthy; it is considerably contracted, and the discharge is moderate. His strength is considerably improved; pulse 116; tongue dry; skin warm. Omitt. vinum. Cap. haustus. salin. ℥j. 2da quaque hora.

25th. Passed a good night. This morning, about half past eight, while sitting up in bed at breakfast, hæmorrhage took place from the wound. On removing the dressing, the bleeding had stopped; a little wine was given, and warm sponges applied to the wound, but the bleeding did not return, although the action of the heart was fully restored. About twelve or fourteen ounces of blood were lost. Pulse an hour afterwards 100.

26th. Passed the day quietly; an injection was given in the evening; complained of burning pain about an inch from the extremity of the rectum, on the introduction of the pipe. Slept well during the night.—About five o'clock this morning hæmorrhage again took place: Mr. Griffiths, assistant-surgeon, who was in attendance on him, finding he could not restrain the bleeding by pressure above or below the clavicle, pushed his finger through the newly-formed granulations, and reached the mouth of the bleeding vessel, by which complete command was obtained over it. It was ascertained to be the axillary artery above the coracoid process. I immediately determined on tying the artery a little below the clavicle. An incision was made from the under part of the clavicle, in the course of the artery, which terminated in the wound. In dissecting down upon the artery, the knife came in contact with a splinter of bone, about half an inch in length, and about a quarter of an inch in breadth, which lay immediately over the artery. By cautious dissection, the artery was separated from the surrounding nerves; and after some difficulty, from the depth and narrowness of the wound, a ligature was passed round it, by insinuating the eyed extremity of a common probe bent, and armed with a ligature under the artery, and drawing the ligature through with a pair of forceps, withdrawing the probe in the same manner in which it entered, leaving the ligature behind. Two small arterial branches were divided, and secured. About half an hour was taken up in the operation, which was performed under disadvantages which those only, who have been similarly situated, can conceive. An open axillary artery; the patient in a moveable cot; considerable motion of the ship; and all this by candle-light. About a pound of blood was lost. The pulse was extremely rapid, and weak; countenance pale and anxious; voice weak. A large draught of tincture of opium was given, and Port wine in small quantity; he complained of urgent thirst. About ten o'clock, he vomited the drink he had taken. At half-past ten o'clock, pulse 144, very weak, and indistinct; sweating copiously from the face; trifling oozing of blood from the recent wound, which scarcely stains the dressing. Habeat vini rubri pauxill. subinde. Sumat jusculum ad libitum.—Two P. M. Has slept; pulse 128, firmer; voice stronger; thirst urgent; complains of pain from the wound. Cont.—Six, P. M. Pulse 120, considerably firmer; has taken strong soup frequently; voice again natural, and countenance cheerful, although very pale.—Eight, P.M. Pulse 124; thirst less urgent. Habeat opii, gr. ij.

27th. Passed the night quietly, although he did not sleep well; slight chills about midnight; pulse 112; brownish discharge from the lower part of the recent wound; granulations of the former wound pale and glassy; little discharge; healthy pus from an opening leading to the glenoid cavity. Cont. vin. tuscul. et haust. anod.—Hora 8ta. P. M. Pulse 116; has taken soup frequently during the day.

28th. Pulse, at six o'clock this morning, 108; slept well the greater part of the night, having had no stool for two days; an enema was ordered this morning, but the introduction of the pipe gave so much pain that it was immediately withdrawn; about seven ounces of pus followed it in a full stream, and afterwards a small quantity of fæces; half an ounce of castor oil was given, which excited nausea and vomiting; soon after had a violent rigor, which lasted about ten minutes, during which the pulse was extremely rapid and irregular.—Hora xii. Pulse 124; heat of skin natural; countenance pale and anxious; alæ nasi dilated at each inspiration. From this time he continued to be affected occasionally with violent rigors, followed by increased heat; little discharge took place from the wound; the ligature continued attached to the artery, and no return of hæmorrhage took place; his strength gradually sunk; he had slight delirium at times, and on the evening of the 3rd of October he expired, without a struggle, eight days after the artery had been tied.

### APPEARANCES AFTER DEATH.

The flap was found firmly united in every part, except over the glenoid cavity; the centre of the cartilage was absorbed—the absorption of the upper half advanced; a sinus was found to extend from the back part of the glenoid cavity under the supraspinatus; the coracoid process and acromion were particularly examined, but they were uninjured. I have no doubt, therefore, that the splinter of bone, the cause of the mischief, must have belonged to another man, especially as at the same time when this patient received his wound many men around him were either killed or wounded. The ligature came away from the artery while examining it; a firm coagulum filled the artery to the extent of about half an inch from the part which had been tied; about the same distance below this part, a round ulcerated hole was found in the coats of the artery; the abdominal viscera appeared healthy, except the sigmoid flexure of the colon and rectum; the former was contracted and thickened, the mucous coat of the latter much thickened and streaked, of a dark purple colour; about an inch from the extremity of the rectum two small openings were found, through one of which a probe could be passed along through the os cocygis, the other led between the rectum and bladder.

Case 2. Charles Taylor, ætat. 26, of a spare delicate habit. A musket ball entered the left side of the thorax, about three inches from the spine, and immediately over the ninth rib, which is fractured; ball lodged; pain in the left side of the epigastric region; breathing quick; frequent cough. Fiat V. S. ad ℥xxiv. cap. sulph. magnesiæ ℥j. Low diet; medicine operated briskly.

29th. Pain in the left hypogastric region; frequent cough; breathing quick; countenance anxious and desponding. Rep. V. S. ad ℥xxvj. aq. supertart. potassæ pro potu.

30th. Was much relieved by the bleeding; pulse 90, soft; breathing easier; frequent cough; bowels free. Potus supert. potassæ.

September 1st. While at stool this morning, a musket ball passed per anum; pulse 90; skin cool; anxious and desponding; has slept little since the action. Cap. haust anod. h. s. rep. potus.

6th. Pulse 100, soft; breathing easy; cough; bowels open; pains in one spot of the abdomen at the lower and left part of the epigastric region; copious discharge from the wound. Rep. potus.

10th. Copious discharge from the wound; breathing easy; little cough; passes sleepless nights, unless when the anodyne draught is given; pulse quick, soft; expresses fears of his recovery frequently, and seems peevish and desponding; bowels slow. Cap. ol. ricini ℥ß.

13th. A piece of cloth removed from the wound this morning. Cont.

14th. *Vespere* hora 6ta. Severe pain in the left side of the abdomen; breathing quick; pulse 130; skin hot. Fiat V. S. ad ℥xxxvj.—Hora 1, A. M. Was but little relieved by the bleeding; pain severe; pulse 120, small. Rep. V. S. ad ℥xvj. Appl. empl. epispast. abdomini dol. part.

15th. Pain much relieved; pulse 124, soft and small; skin warm and moist; copious thin discharge from the wound; bowels open. Cont.

16th. Pulse 100, soft; the posterior part of the fractured rib rises over the anterior part; copious discharge; no appearance of granulations. From this period the pulse seldom fell below 100; he continued to be affected with slight cough, occasional pain in the left side of the epigastrium, and night sweats; the wound became fistulous, and the carious* portion of the fractured rib remained firmly fixed, when he was sent to Hospital on the 8th of October.

It would be idle to conjecture at the exact course which the ball took in this case, although it can scarcely be doubted that the lungs were wounded, as well as the intestinal canal; yet the absence of emphysema, and the comparatively slight affection of the breathing, may raise a doubt with some on this head. But it must be recollected, that emphysema by no means necessarily follows wounds which have unequivocally injured the lungs. I have little doubt that the ball made its way into the descending colon; although some were persuaded, from the apparent direction of the wound, that it had entered the stomach. The freedom, however, from symptoms which attend wounds of this most delicate organ renders this improbable.

Case 3. August 28th. Samuel Norman, marine, ætat. 35, of a robust habit, was struck by a large shot on the left side of the thorax, immediately under the axilla; the ribs were fractured without external wound of the integuments; extensive ecchymosis; emphysema of left side;

---

* Since this essay was drawn out, I have perused the very valuable work of Mr. Henning, in which notice is taken of this Case, page 383. His account of the Case will be found in some respects incorrect.

breathing much oppressed. Fiat V. S. ad ℥xxx. Capt. cath. et applic. fascia thoraci.

29th. Breathing relieved by the bleeding; it is much less oppressed; emphysema does not extend; frequent cough; he is supported in bed, in a half erect posture, the position in which his breathing is easiest; pulse 96. Fiat V. S ad ℥xvj. aq. supert. potassæ ad libit.—*Vespere.* Breathing more laborious. Rep. V. S. ad ℥xxx.

30th. Breathing laborious; conntenance anxious; pulse quick, and of sufficient strength; skin hot. Rep. V. S. ad ℥xx. Emphysema does not extend. *Vespere.* Was relieved by the bleeding; breathing still oppressed. Rep. V. S. ad. ℥xvj.

31st. Breathing considerably easier; pulse 100, and soft; frequent cough; bowels open; emphysema does not extend. Cont. potus; appl. fotus aceti lateri.

September 6th. Breathing has continued much easier, emphysema and ecchymosis disappearing; pulse 90, and soft; skin cool; bowels open. Cont. potus et fotus.

From this period his recovery advanced regularly; the ecchymosis, emphysema, and cough gradually disappeared, and on the 17th of Sept. he was free from complaint.

Case 4. August 28th. Thomas Freeman, marine, ætat. 20. A musket ball entered the abdomen, about three inches from the superior anterior spinous process of the ilium, and above it, and escaped about an equal distance from the spinous process on the left side; frequent vomiting and constant nausea; pulse 90. Fiat V. S. ad ℥xx. Inj. enema.

29th. General pain in the abdomen; frequent vomiting; pulse 90, and contracted; skin cool. Rep. V. S. ad ℥xxviij. No stool. Inj. enema purgans statim, et app. fotus callid. abdomini frequenter; potus supert. potassæ.

30th. Several stools yesterday; singultus at times; pain of abdomen relieved; vomiting less frequent; pulse 96, small; skin cold. Rep. enema et fotus, cap. haust. æther. pro singultu.

31st. Wounds easy; pain of abdomen relieved; four stools; singultus less frequent. Cont.

September 1st. Singultus more troublesome; increase of pain of the abdomen; pulse 110, small, but firm. Rep. V. S. ad ℥xxx.

2nd. Was greatly relieved by the bleeding; still complains of pain about the umbilicus; nausea and vomiting at times; no singultus; sweated profusely last night; pulse 90, and small; skin very cool; one stool. Inj. enema, rep. fotus et cap. haust efferves. subinde.

3rd. Pain of abdomen much easier, but it is distended; pulse 76, soft; tongue furred; sloughs of the wounds separating; no nausea or vomiting. Cap. sulph. magn. ℥vj. si opus sit, inj. enema. Rep. potus et fotus. — *Vespere.* Pain of abdomen increased; it continues tense and swelled; two stools. Rep. V. S. ad ℥xvj.

4th. No complaint this morning but of pain about the wounds; abdomen continues tense and swollen; pulse 80, soft; skin cool; tongue slightly furred; expresses a desire for food; not granted. Rep. enema et fotus.

5th. Swelling and tension of abdomen much abated; no pain but about the wounds; slept well; pulse 80. Rep. ut heri.

On the 16th of September he had a slight attack of bilious vomiting, and scanty bilious stools. Calomel in moderate doses was administered until the 22nd, when the stools became natural. On the 24th. both wounds were healed; a slight induration remaining at that on the left side, which gradually disappeared, and, on our arrival in port, he had no complaint but of slight debility.

It may be doubted whether the ball penetrated the cavity of the abdomen in this case, from the absence of those symptoms which unequivocally denote a wound of this cavity—the protrusion of some of the bowels, or the escape of their contents by the wound.

In wounds from musket balls, which only penetrate the parietes of the abdomen, we are always able after inflammation has come on, to trace the course of the ball by the hardness in its track, and by a blush of inflammation on the integuments covering its course. These were not apparent in this case. The symptoms, at all events, sufficiently marked the existence of inflammation of the abdominal viscera, which the treatment employed fortunately subdued.

Case 5. George Thomson, ætat. 19, received a wound over the base of the right scapula, immediately below the spine of the bone, which had the appearance of a wound from a large grape-shot. The penetrating substance had taken a course across the scapula, which it had shattered to pieces. A hard body was perceived projecting over the upper part of the posterior fold of the arm-pit; it was cut down upon, and found to be a portion of the fractured scapula. Many fragments of bone were removed from the wound. A few days afterwards, a hard substance was perceived in the incision made on the back part of the shoulder, which could be traced under the deltoid muscle and acromion, and covering the back and forepart of the shoulder-joint. Two extensive incisions were required for its removal; and it was found to be a large splinter of a shell, which weighed about seven ounces. Many portions of the scapula were removed at different times from the wound; his health continued good; the wound required for the extraction of the foreign body healed, except a small point from which the under part of the acromion could be felt bare at the time he was discharged to the hospital, on the 8th day of October.

This case is only interesting as it shews how a foreign substance of considerable magnitude may be lodged in the body for a time, without affording any marks by which its situation may be detected.

It has been a question of late, which has caused some discussion, at what period, after the receipt of a wound, amputation ought to be performed in cases which decidedly require the operation? It is now, I believe, agreed upon by all surgeons, who have had sufficient opportunity of observation, and of judging of the merits of the case, that, on no occasion ought the operation to be deferred until suppuration is established; for, so far from this state diminishing the risk from the operation, as was formerly believed, the more than sufficient experience on this point which the events of the late war have afforded incontestably prove, that of given numbers, on whom secondary and primary amputation are performed, a much smaller proportion of the former recover than of the latter; leaving entirely out of account the many cases which never arrive at the proper period for amputation; of those on whom the operation has been deferred; and many of whom, it may reasonably be supposed, the primary amputation would have saved.*

The question now at issue is, whether, when the necessity of amputation is evident, we ought immediately to have recourse to the operation; or whether we ought to wait for a time, longer or shorter, according to the circumstances of the case, until the patient has recovered from a state of general nervous commotion which, the advocates for a short delay say, generally follows severe injuries requiring amputation, and which increases greatly the danger of the operation, if performed under this state. I am clearly of opinion, that where we are decided on the necessity of amputation, the sooner the shattered limb is

* See Guthrie's valuable Work on Wounds.

removed the better; and this state of commotion, if it did take place, would be a farther inducement to me to proceed to the operatlon, without a moment's delay. This state of commotion must undoubtedly arise from the irritation communicated from the torn parts; and while they remain attached to the body, we can scarcely hope that the effect will cease. Mr. Guthrie says, at page 51 of his work, "*There is, in general, a certain period differing in different persons, as to time, before these symptoms of commotion come on, and they are then severe in a greater or less degree*". If these symptoms arose from the immediate shock of the wound, we should surely expect them to follow immediately the infliction of the injury; but as a certain period intervenes between their appearance and the injury, it may be more reasonable to attribute them to the irritation communicated from the torn parts. Any approach, however, to this alarming state of commotion I would say, from my own very limited experience, was a rare occurrence. I saw nothing like it in any of the cases requiring amputation in the battle of Algiers, except in Case No. 1; and I can scarcely here apply the term of nervous commotion, in the sense in which it is generally received when applied to cases of this kind; for although the body was evidently suffering from the great loss of blood, and acute anguish from the wound, his mind was calm and determined. The immediate consequences of the operation, in this case, were of the most satisfactory nature, and the circumstances under which it was performed, could scarcely have been more unfavourable. In conversing with the patient, some time afterwards, on the subject of the operation, he expressed himself in the strongest terms of the immediate relief he experienced from it. In his own emphatic manner he said, "I WAS IN ELYSIUM IMMEDIATELY IT WAS OFF."

In cases of this kind, the advocates for delay allow, that the operation must be performed immediately, and it in general brings immediate relief, even if the patient should sink afterwards; from whence this relief, but from the removal of the cause exciting the symptoms? And if, in cases of this severity, where the commotion is in the highest degree, amputation brings immediate relief, and is the only chance the patient has of being saved, surely, cases where the injury is slighter, and where the commotion may be expected to be less, cannot be followed with perfectly opposite effects. As a certain period elapses before the approach of this dreaded commotion, may we not expect to anticipate its appearance by the removal of its cause? I am, therefore, of opinion, that it ought to be laid down as a general rule of surgical practice, that where the necessity of amputation, in consequence of injury, is apparent, it ought to be performed without delay. But there are circumstances in which, I think, this law, in some cases, must be dispensed with; these circumstances, however, do not militate against the general law, as a rule of practice; nor is it for any prospect of advantages which the sufferers are to receive from the operation being delayed, that it is to be put off for a time.

During the continuance of a battle we must, at all times, be ready to afford assistance to the newly wounded, who are every moment requiring it. If, therefore, during the time we are engaged in operating, a bleeding patient should make his appearance, we must either leave the operation unfinished, to afford succour in this urgent case, as, before we have finished, our patient might be lost. In cases, such as No. 1, all other considerations must be laid aside, and the operation must be performed instantly; but where the injury is of a less serious nature, although still with as much certainty of ultimately requiring amputation, I would defer it until after the action. And the history of the amputations performed by me on this occasion, will prove that no subsequent danger is to be apprehended from this delay.

I had determined, previous to the action, from the above considerations, not to operate during its continuance, unless in urgent cases, where the immediate removal of the shattered limb could afford the only chance of saving the patient. But the duration of this battle so much exceeded what usually happens in sea fights, and what had been expected, that after it had continued about six hours, and without a prospect then of its being ended, I proceeded to operate. Seven amputations were performed. One immediately after the receipt of the wound; four from three to six hours after they were wounded; and two about eighteen hours afterwards. The necessity of the removal of the limbs, in these two cases, was sufficiently evident; but the men themselves would not consent to the operation sooner. These cases succeeded equally well as the others. In one of those operated upon last, whose thigh was amputated, the symptomatic fever ran higher than in the others; the stump, however, healed nearly by the first intention.

From the issue of these cases it is evident, that no great danger is to be apprehended from delaying amputation for a short time, (no urgent symptom in the mean time occurring to require it) provided it is performed before symptoms of inflammation or of general reaction have come on. And the fate of naval combats is, in general, so speedily decided, that I would, on such occasions, defer the operation in those cases, where it could be done without immediate risk, until the battle was ended. But it is only in such circumstances, that I would postpone the removal of a shattered limb for an instant; for, during this delay, our patient is suffering considerable pain and distress, when we at least have it in our power to relieve him, by the removal of their cause.

It may, perhaps, be worthy of remark, that of the great number wounded in the Fleet, amounting to about 700, no case of tetanus occurred, although the battle took place at a season of the year, when this disease most frequently shews itself, and the nature of the wounds received, and the circumstances of the patients confined on board ships, seemed particularly to favour its appearance. It is probable the immunity from this dreadful disease may, in some degree, be attributed to the treatment employed, especially to the constant attention paid to the free evacuation of the intestinal canal.*

Although not immediately connected with the subject of this Essay, the unusual good state of health of the crew, during this short, but momentous expedition, may be worthy of notice. The crew of this ship consisted of nearly 1000 men, thrown together hastily for the occasion. Two men were sent to Gibraltar Hospital on our way to the scene of action; one with violent inflammatory affection of the thoracic viscera, and who died a few days afterwards; and one with mania. No one died on board from disease, and no serious case existed on our arrival in England. This high state of health, I have no doubt may, in a great degree, be attributed to the general state of mental excitement kept up previous to the battle, from the moral certainty of its taking place; the constant preparations for it; and the state of exhilaration resulting from the perfect success of the enterprise. Had the excessive exertion required during this dreadful fight been followed by an unsuccessful issue, I have no doubt the account of health would have been widely different. A. DEWAR, M. D.

Stirling, Dec. 1818.

---

* *See* Dr. Dickson's admirable Paper on this Subject, in the Medico-Chirurgical Transactions.

